# Don’t Miss Syphilis: A Mimicker of Lymphoma in an Era of Rising Incidence

**DOI:** 10.7759/cureus.90029

**Published:** 2025-08-13

**Authors:** Atsushi Isoda, Hirono Iriuchishima, Akio Saito

**Affiliations:** 1 Department of Hematology, Iryohojin Hoshiiin, Maebashi, JPN; 2 Department of Hematology, National Hospital Organization Shibukawa Medical Center, Shibukawa, JPN

**Keywords:** differential diagnosis, inguinal lymphadenopathy, lymphoma mimicker, selection bias, sexually transmitted infection, soluble interleukin-2 receptor, syphilis

## Abstract

This report describes the case of a middle-aged man who presented with bilateral inguinal lymphadenopathy and an elevated serum level of soluble interleukin-2 receptor (sIL-2R), initially raising concern for malignant lymphoma. In Japan, sIL-2R is widely reimbursed as a diagnostic adjunct for lymphoma, which heightened the suspicion of malignancy in this case. However, serologic testing for syphilis returned positive, leading to a diagnosis of active syphilis. Lymph node biopsy was deferred, and the patient was treated with oral ampicillin, resulting in the resolution of lymphadenopathy and normalization of sIL-2R levels. This case underscores the importance of considering syphilis in the differential diagnosis of unexplained lymphadenopathy, particularly in regions where incidence is increasing.

## Introduction

Syphilis is a sexually transmitted infection caused by *Treponema pallidum*, and its clinical manifestations are diverse and often nonspecific [1]. Recent years have seen a global increase in reported cases, with a rapid rise also observed in Japan [2,3]. Lymphadenopathy is a well-recognized sign of early syphilis; however, its clinical significance may be overlooked when classic mucocutaneous findings are absent [4]. Serum soluble interleukin-2 receptor (sIL-2R) level is widely used in Japan as a biomarker for lymphoproliferative disorders [5], but it is nonspecific and can be elevated in infectious and inflammatory diseases [6-9]. In the present case, the patient was initially suspected of having lymphoma based on elevated sIL-2R levels and imaging findings, but was ultimately found to have syphilis. This case underscores the importance of maintaining a broad differential diagnosis, as syphilis can convincingly mimic malignant disease and mislead clinicians, even when laboratory and imaging findings are suggestive.

## Case presentation

A 50-year-old man presented to his primary care physician with a two-week history of painless bilateral inguinal swelling. He denied fever, night sweats, weight loss, fatigue, or other constitutional symptoms. Physical examination revealed multiple non-tender, mobile lymph nodes, each approximately 3 cm in diameter, in both inguinal regions. No abnormalities were noted in the external genitalia, perianal region, or oral cavity. There were no skin rashes, including palmar or plantar lesions, and no oral ulcers or neurological deficits. Infectious lymphadenitis was initially suspected, and moxifloxacin was prescribed; however, there was no improvement.

**Figure 1:**
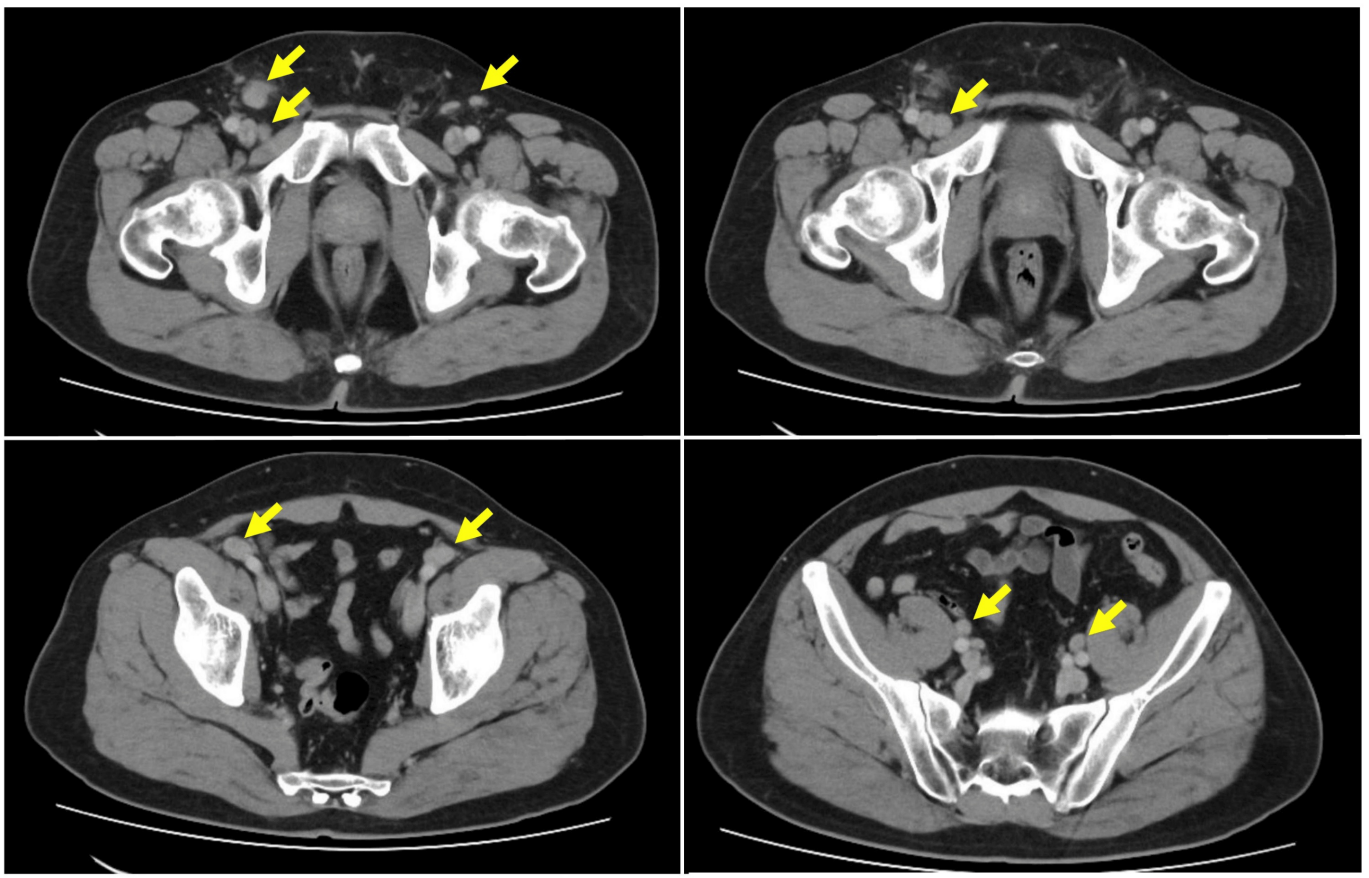
Contrast-enhanced CT scan showing lymphadenopathy along the bilateral inguinal and femoral vessels. The lymph nodes exhibited homogeneous internal architecture, with no evidence of necrosis or calcification. Arrows indicate the regions of interest.

Laboratory tests revealed normal blood counts and lactate dehydrogenase, but an elevated sIL-2R level of 1,438 U/mL, prompting referral to a hematology center for evaluation of possible lymphoma (Table 1). Contrast-enhanced CT showed lymphadenopathy along the inguinal and femoral vessels, with no other remarkable findings (Figure 1). Lymphoma remained a leading differential diagnosis, and a lymph node biopsy was planned.

**Table 1:** Laboratory results on first presentation. WBC: white blood cell; RBC: red blood cell; TP: total protein; T-Bil: total bilirubin; AST: aspartate aminotransferase; ALT: alanine aminotransferase; LDH: lactate dehydrogenase; ALP: alkaline phosphatase; BUN: blood urea nitrogen; CRP: C-reactive protein; SIL-2R: soluble IL-2 receptor; IgG: immunoglobulin G; IgA: immunoglobulin A; IgM: immunoglobulin M

Variable (unit)	Patient value	Reference range
WBC (/μL)	7,100	3,900–9,800
Neutrophil (%)	59	30–78
Lymphocyte (%)	29	18–60
Monocyte (%)	10	3–10
Eosinophil (%)	2	0–5
Basophil (%)	1	0–1.2
RBC (×10^6^/μL)	4.72	4.27–5.20
Hemoglobin (g/dL)	13.4	13.5–17.6
Hematocrit (%)	41.5	39.8–51.8
Platelets (×10^4^/μL)	31.9	13.1–36.2
TP (g/dL)	7.9	6.7–8.3
T-Bil (mg/dL)	0.54	0.3–1.2
AST (U/L)	27	13–33
ALT (U/L)	57	8–42
LDH (U/L)	158	119–229
ALP (U/L)	204	115–359
BUN (mg/dL)	10.7	8–22
Creatinine (mg/dL)	0.86	0.6–1.1
Na (mEq/L)	138	138–146
K (mEq/L)	4.6	3.6–4.9
Cl (mEq/L)	102	99–109
CRP (mg/dL)	1.89	0–0.14
Anti-nuclear antibody	<1:40	<1:40
SIL-2R (U/mL)	1,438	156–474
IgG (mg/dL)	1,698	861–1,747
IgA (mg/dL)	553	93–393
IgM (mg/dL)	102	33–183

However, as part of a pre-biopsy infection screening, serologic testing was positive for rapid plasma reagin (RPR; latex turbidimetric immunoassay, 18.0 RU) and *Treponema pallidum* hemagglutination assay. This was the patient’s first syphilis screening. HIV and hepatitis B virus serologies were negative. The mildly elevated C-reactive protein (1.89 mg/dL) and alanine aminotransferase (57 U/L) were considered consistent with systemic inflammation and mild hepatic involvement occasionally observed in secondary syphilis. The patient recalled a single episode of oral sex with a non-regular partner three months earlier, without any noticeable rash or genital ulcer. Based on these findings, he was diagnosed with secondary syphilis and received a six-week course of oral ampicillin, an alternative regimen employed in Japan when intramuscular benzathine penicillin G is unavailable [10].

Following treatment, the RPR titer declined to 4.0 RU at three months, the lymphadenopathy resolved completely, and the sIL-2R level returned to normal. The biopsy was canceled, and no recurrence was observed at the 10-month follow-up.

## Discussion

This case demonstrates that syphilis can closely mimic malignant lymphoma, particularly when classical mucocutaneous findings are absent. Bilateral inguinal lymphadenopathy and elevated sIL-2R initially raised strong concern for a lymphoproliferative disorder. In Japan, sIL-2R is widely reimbursed and routinely used as a diagnostic adjunct for non-Hodgkin lymphoma, and it is commonly measured in the evaluation of lymphadenopathy [5]. However, because sIL-2R is not disease-specific and reflects T-cell activation, elevations can occur in a wide range of conditions, including tuberculosis, Epstein-Barr virus infection, HIV, and syphilis [6-9]. A Japanese cross-sectional study further highlighted that the optimal cutoff value for diagnosing malignant lymphoma varies according to febrile status [11]. Therefore, clinical interpretation of sIL-2R must be contextual. In the present case, syphilis initially acted as a diagnostic pitfall; however, pre-biopsy serologic screening allowed for the correct diagnosis and avoided unnecessary invasive intervention.

Although uncommon, syphilis may present predominantly with lymphadenopathy in the absence of characteristic mucocutaneous lesions, sometimes prompting suspicion of malignancy. Van Crevel et al. described two cases of isolated cervical syphilitic lymphadenitis without obvious sexually transmitted infection risk factors [12]. Yorita et al. reported primary syphilis manifesting solely as painful unilateral inguinal lymphadenopathy without skin involvement [13]. Other reports have documented secondary syphilis with generalized or regional lymphadenopathy initially suspected to be lymphoma, with the correct diagnosis made only after serologic testing or histopathology [14-16]. These observations align with our patient’s presentation and highlight the need to include syphilis in the differential diagnosis of unexplained lymphadenopathy.

This case also highlights the role of selection bias in clinical decision-making. In Japan, where the incidence of syphilis has long been considered low, these infections are often omitted from the differential diagnosis in primary care. In contrast, patients referred to secondary or tertiary care are typically selected based on worrisome features such as antibiotic-refractory lymphadenopathy or elevated tumor markers, fostering a diagnostic bias toward malignancy. The widespread use of sIL-2R may further reinforce this bias, potentially leading to underrecognition of benign or infectious causes.

**Figure 2:**
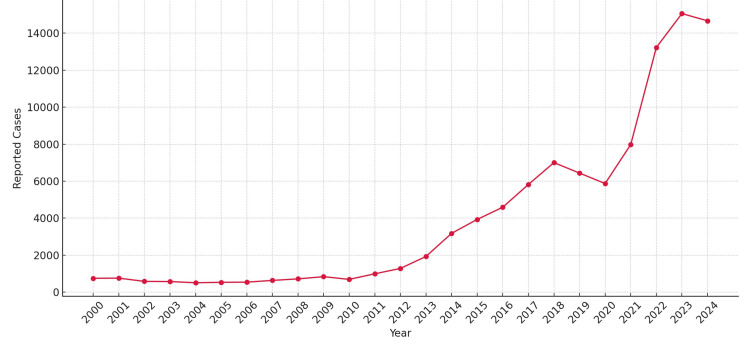
Annual reported cases of syphilis in Japan from 2000 to 2024. The number of syphilis cases remained under 1,000 per year in the early 2000s but began to rise significantly after 2013, reaching over 15,000 cases in 2023. This upward trend has continued, with more than 14,000 cases already reported in 2024 (provisional data). Data derived from the National Institute of Infectious Diseases (NIID), Japan [18].

In 2024, over 14,000 cases of syphilis were reported in Japan (Figure 2), aligning with trends documented by the World Health Organization and Centers for Disease Control and Prevention [3,17]. Notably, the post-COVID-19 resurgence in Japan has outpaced increases observed in other regions, and infections have been increasingly reported even among individuals without clearly defined sexual risk factors [2]. Reduced public health center testing during the pandemic may have contributed to missed diagnoses, delayed treatment, and further transmission. These observations underscore the importance of maintaining a high index of suspicion for syphilis, regardless of apparent risk behaviors. This case also highlights the limitations of relying solely on patient-reported sexual history. Social stigma and a lack of awareness regarding early symptoms often result in underreporting or omission during history-taking. Therefore, in young to middle-aged adults presenting with unexplained lymphadenopathy, routine screening for sexually transmitted infections, including syphilis, acute HIV infection, *Chlamydia trachomatis*, and *Neisseria gonorrhoeae*, should be actively considered.

This case also underscores the value of a stepwise, minimally invasive diagnostic approach in the evaluation of unexplained lymphadenopathy [19,20]. Early serologic screening, in particular, can help avoid unnecessary invasive procedures and overdiagnosis, while reducing patient anxiety and conserving healthcare resources. When infectious causes of lymphadenopathy are overlooked, diagnostic delays may result in postponed treatment, disease progression, or increased risk of transmission. Therefore, timely serologic evaluation with infectious etiologies in mind is essential for guiding prompt and appropriate therapy.

Ultimately, this case highlights syphilis as a potential mimicker of malignancy and draws attention to broader diagnostic challenges, including cognitive bias, overreliance on biomarkers such as sIL-2R, and the evolving epidemiology of infectious diseases. In an era of shifting disease patterns, diagnostic flexibility and heightened awareness remain crucial.

## Conclusions

Syphilis remains a crucial consideration in the differential diagnosis of lymphadenopathy. Particularly in countries like Japan, where incidence is increasing, syphilis should not be overlooked. Elevated sIL-2R levels do not always indicate malignancy, and infectious etiologies must be considered. This case demonstrates that a combination of clinical history and appropriate serologic testing can prevent misdiagnosis and avoid unnecessary invasive procedures. Clinicians should remain alert to syphilis as a potential masquerader of malignancy, especially in the context of unexplained lymphadenopathy.
